# Perinatal brain growth and autistic traits in toddlers

**DOI:** 10.1038/s41398-025-03665-0

**Published:** 2025-11-17

**Authors:** A. Tsompanidis, K. M. Chang, Y. T. Khan, M. A. Radecki, L. Dorschmidt, S. Hampton, E. Aydin, C. Allison, R. Tait, R. A. I. Bethlehem, V. Kyriakopoulou, T. Austin, J. Suckling, R. Holt, S. Baron-Cohen

**Affiliations:** 1 Autism Research Centre, Department of Psychiatry, University of Cambridge, Cambridge, UK; 2 Social and Affective Neuroscience Group, IMT School for Advanced Studies Lucca, Lucca, Italy; 3 Department of Psychiatry and Lifespan Brain Institute, University of Pennsylvania, Pennsylvania, USA; 4 York Trials Unit, University of York, York, UK; 5 Vagelos College of Physicians and Surgeons, Department of Psychiatry, Columbia University, Cambridge, USA; 6 Department of Psychology, University of Cambridge, Cambridge, UK; 7 Cambridge Open Zettascale Lab, University of Cambridge, Cambridge, UK; 8 Department of Early Life Imaging, School of Biomedical Engineering and Imaging Sciences, King’s College London, St Thomas’ Hospital, London, UK; 9 NeoLab, Evelyn Perinatal Imaging Centre, Rosie Hospital, CUH NHS Trust, Cambridge, UK; 10 Psychiatric Neuroimaging, Department of Psychiatry, University of Cambridge, Cambridge, UK

**Keywords:** Predictive markers, Human behaviour

## Abstract

Autism is a heterogeneous set of neurodevelopmental conditions with a significant heritable component and perinatal origins. The earliest observable behavioural traits, with which a diagnosis can be made, emerge at 18 months of age. Previous studies have reported increased head circumference and brain growth in autistic children, but less is known about whether this extends to the wider spectrum of traits or to early brain development in the first 6 months of life. Data from two independent cohorts, the developing Human Connectome Project (dHCP) and Cambridge Human Imaging and Longitudinal Development (CHILD), were assessed in late fetal and early infancy for brain structure with MRI. Global and regional brain volumes in the dHCP, were studied prenatally (n = 106, mean age = 29.27 [SD = 3.8] gestational weeks) and postnatally (n = 454, mean age = 41.28 [SD = 1.93] weeks post-conception) in association with later autistic traits after 18 months of age, as captured on the parent-report Quantitative Checklist for Autism in Toddlers (‘Q-CHAT’). The results informed a region-of-interest analysis in the smaller independent CHILD cohort, where participants were scanned during pregnancy (mean = 31.98 weeks gestation, SD = 1.52) and postnatally (mean scan age = 50.45, SD = 2.8 weeks post-conception)(n = 27). After controlling for cohort covariates, such as maternal age, birth weight, sex and age post-conception at the time of scan, postnatal total brain volume, cortical grey matter volume, and white matter volume were all negatively associated with autistic traits in toddlerhood. This was found for postnatal volumes in both the dHCP and the CHILD cohort but was not apparent when assessing prenatal brain volume or perinatal growth rates of total brain volume in the same individuals. Regional analyses in the dHCP cohort, after controlling for total brain volume, showed patterns of note in the temporal lobe, which warrant further research. In conclusion, reduced total brain volume in the first two months of life is associated with a higher number of autistic traits, as reported by parents at 18 months of life. Further research is required to understand if this extends to later ages, to children later diagnosed with autism and how it affects the development and connectivity of specific regions, particularly in the temporal lobes.

## Introduction

Autism is a set of neurodevelopmental conditions that involves difficulties in social interaction and communication and adjusting to unexpected change, alongside unusually restricted interests and repetitive behaviours and sensory hyper-sensitivity. Based on twin-heritability estimations, genetic sequencing and genome-wide-association studies, there is now consensus that most of the genetic influences in autism (diagnosis or traits) can be attributed to genetic variance [1, 2].

A diagnosis of autism often occurs after 18-months of age, when specific neurodevelopmental behaviours emerge in toddlerhood. These traits pertain to social attention, language use, and the development of cognitive skills such as ‘theory-of-mind’. The Quantitative Checklist for Autism in Toddlers (Q-CHAT) is a psychometric measure, rated by a parent or caregiver, that was developed to capture these traits in an additive way [3]. Since the Q-CHAT was first reported, several studies have confirmed its validity as a screening tool that predicts a later autism diagnosis in children following gold-standard neurodevelopmental assessments by specialists (i.e., based on DSM-IV/5 or ICD-10 criteria). Validation studies for the Q-CHAT have been conducted in different populations of different ethnicities, with consistent results [4–6].

Previous studies that examined autistic traits as a continuous outcome in the general population reported varying patterns of association to brain structure. These included mostly global brain measures, with negative correlations between autistic traits and intercranial volume [7], total brain volume or cerebellar volume in childhood [8, 9]. Interestingly, associated patterns were independent to autism genetics [8, 9], remained stable over time [9] and extended to individuals with traits below the diagnostic thresholds for autism [8]. Similar studies have also found associations of traits with the volume in subcortical structures, as well as with white matter integrity [7, 10]. So far, no study has explored whether the earliest autistic traits in toddlerhood are associated with total or regional measures of brain structure, and, specifically, with patterns of perinatal brain growth.

On the other hand, in children diagnosed as autistic, retrospective assessments of their brain development have reported evidence of global ‘brain overgrowth’ compared to non-autistic children [7, 11–14], with the earliest indication of differences becoming statistically significant after 6 months of age [15]. A similar observation has been reported when estimating the total number of neurons, with autistic males having more neurons than undiagnosed males [16]. Yet, evidence in larger cohorts or when assessing fetal head circumference, remains mixed [17, 18]. In fact, a subset of autistic people have been found to have early brain undergrowth, particularly before 6 months of age 7.

Several aspects of “brain overgrowth” in autism remain unclear, including whether (a) it starts earlier than 6 months of age, (b) affects some brain regions more than others, and (c) extends to individuals with a high number of autistic traits in the general population.

**Fig. 1:**
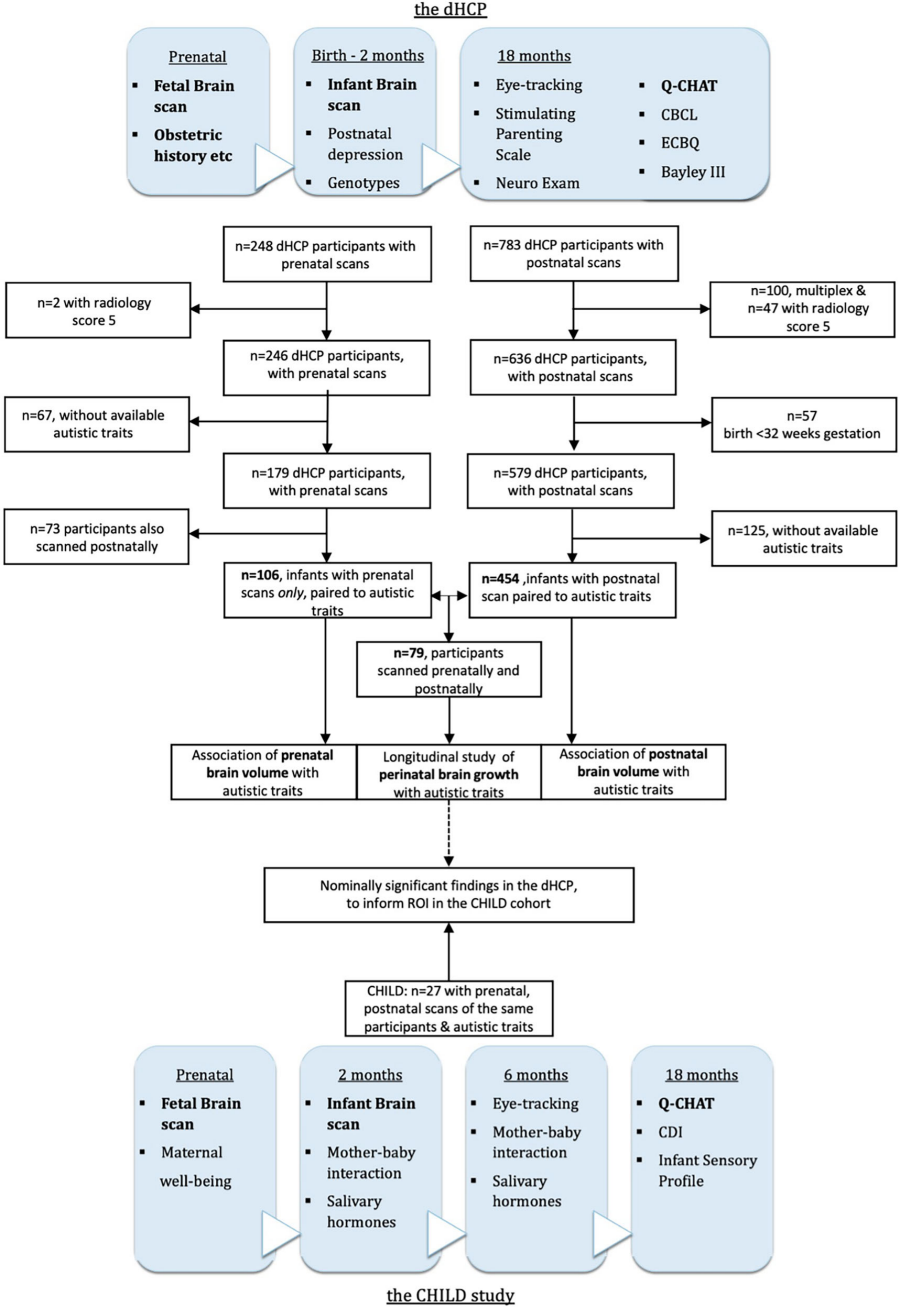
Study flowcharts including the basic prospective design of the dHCP (top) and CHILD study (bottom). Items in bold are featured in this study. “Nominal significance” refers to uncorrected p-value of less than 0.05. Q-CHAT quantitative checlist of autism in toddlers, CBCL Child Behaviour Checklist, CDI Communicative Development Inventories, ECBQ Early Childhood Behaviour Questionnaire.

To address these questions, in the present study two independent cohorts were assessed in terms of perinatal brain development via MRI and their autistic traits in toddlerhood as captured by the Q-CHAT; namely the participants of the developing Human Connectome Project (dHCP, n = 454) scanned soon after birth, and the participants of the Cambridge Human Longitudinal Development study (CHILD, n = 27)(Fig. 1). Both cohorts are prospective, including prenatal and postnatal MRI brain scans. Both studies include measures of autistic traits as an outcome at 18 months (via the Q-CHAT), with the CHILD cohort also being enriched with participants from families with a family history for diagnosed autism (presence of an autistic parent or sibling).

## Results

### Q-CHAT score variance and cohort characteristics

**Fig. 2:**
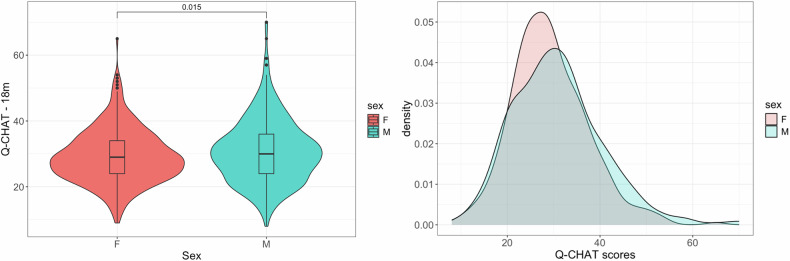
Autistic traits. In the dHCP, males had higher autistic traits on average, as measured by the Q-CHAT at 18 months. Left panel: p-value is from student’s t-test.

In the dHCP cohort, Q-CHAT scores were available for a total of n = 744 participants (mean Q-CHAT = 30.06 (SD = 9.07). Toddlers assigned as male at birth had significantly higher scores on the Q-CHAT than toddlers assigned as female (Student’s t-test: t = −2.44, p = 0.015, Cohen’s D = 0.177) and this was also confirmed in linear regression models that were controlled for parental ages, gestational age at birth and the age of the infant at the time of assessment (Fig. 2). There was a trend for an effect of prematurity, with Q-CHAT scores being higher in toddlers born extremely preterm (n = 26, <28 weeks gestation), compared to toddlers born at term (Student’s t-test: t = −1.99, p = 0.058, Cohen’s D = 0.55). When splitting the sample by sex, this effect was statistically significant for females (n = 14 extremely preterm, Student’s t-test: t = −2.87, p = 0.014, Cohen’s D = 1.00) but not for males (Supplementary Fig. 1). When assessed as a continuous variable, gestational age at birth was significantly and negatively associated with Q-CHAT scores Pearson’s r = −0.08, p = 0.026). In addition, Q-CHAT scores correlated negatively with maternal age (Pearson’s r = −0.08, p = 0.026) and birth weight (Pearson’s r = −0.09, p = 0.023), but not with paternal age or parity (assessed linearly via Pearson’s correlation and with pairwise t-tests) (Table 1) (Supplementary Fig. 2).

**Table 1:** Continuous cohort characteristics in the entire dHCP cohort, and their association with Q-CHAT scores in pairwise Pearson’s correlations.

	Mean (SD)	Effect size for Q-CHAT	95% CI	p-value
Maternal age	34.15 (4.57)	r = −0.081	−0.152–−0.010	0.026*
Paternal age	36.47 (6.16)	r = 0.004	−0.070–0.077	0.923
Maternal parity	n−0 = 487	r = 0.034	−0.038–0.106	0.353
	n−1 = 179			
	n−2 = 49			
	n−3 = 17			
	n−4 = 6			
	n−5 = 1			
Gestational age at birth	37.1 (4.68)weeks	r = −0.104	−0.178–0.031	0.006*
Birth Weight	2.98 (0.90) kg	r = −0.09	−0.170–−0.013	0.023*
Age at Q-CHAT	19.8 (2.60) months	r = −0031	−0.103–0.040	0.392

Asterisk denotes significance at.

*p < 0.05.

In the CHILD cohort, Q-CHAT scores were available for n = 36 infants, as assessed at 18 months of age (mean = 32.4, SD = 10.5). There was no significant difference between males (n = 19) and females (n = 17) (Student t-test: t = 0.91, p = 0.372), or with any of the other assessed cohort demographics, including maternal age (Supplementary Table 2). Infants of families with a history of autism (parent or sibling) scored significantly higher (n = 10, mean= 41.6, SD = 10.1) than infants with no family history of the condition (n = 26, mean = 28.9, SD = 5.7, Student’s t-test = 2.65, p = 0.024, Cohen’s D = 1.41).

### Postnatal global brain metrics and autistic traits in the dHCP cohort

For the purposes of this study and to avoid potential volumetric outliers, MRI scans from the dHCP were excluded if they corresponded to infants from multiplex pregnancies, from infants born very or extremely preterm (before 32 gestational weeks, at birth) and in cases where the radiology score for the scan was 5, indicating atypical patterns of clinical and research significance as rated by a specialised perinatal neuroradiologist (e.g., white matter injury, ventricular dilatation, cortical folding abnormalities, cystic lesions or intracranial haemorrhages/infarcts) (Fig. 1). In cases where more than one postnatal brain scan was available (n = 26), only the second scan was included in subsequent analyses, to better approximate the developmental time-point corresponding to typical duration of term (cohort mean = 41.23 weeks, SD = 1.93).

**Fig. 3:**
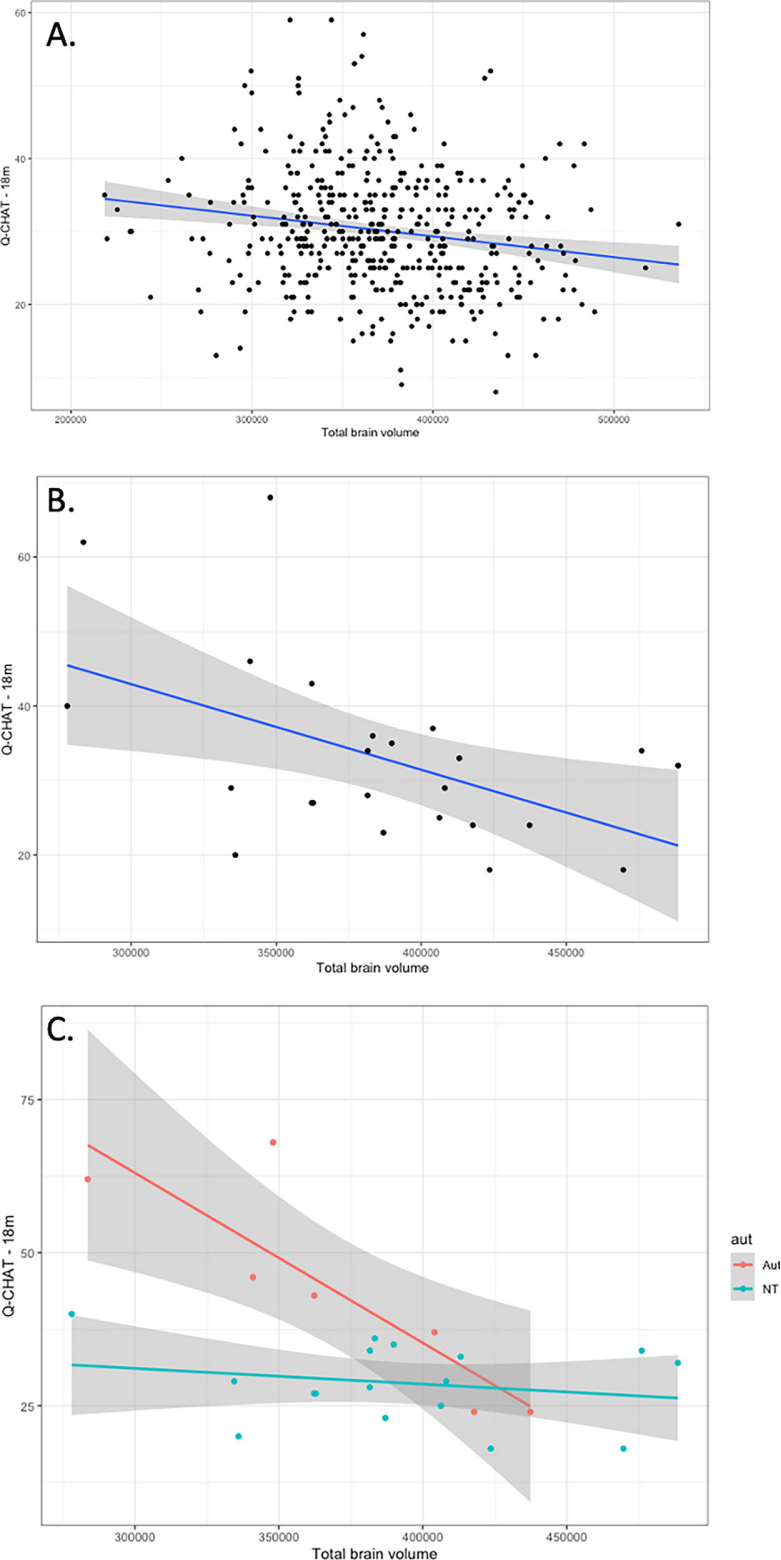
Brain volume and autistic traits. Total brain volume in the first two months of life was associated with autistic traits in (**A**) the dHCP cohort (**B**-**C**) CHILD cohort, in which the effect was driven by individuals with a family history of autism (interaction shown in C).

In this subset of the dHCP cohort (n = 454), infant total brain volume (TBV) was negatively associated with autistic traits, as assessed on the Q-CHAT at 18 months (Fig. 3A), in a model that also controlled for sex at birth, maternal age, birth weight and age post-conception at the time of MRI scanning and age of Q-CHAT assessment (Table 2). A negative association was also found between Q-CHAT scores and total grey and white matter in the same individuals and using the same model covariates, but this was not significant for intracranial CSF (cerebrospinal fluid) volume.

**Table 2:** Multiple regression models of total brain metrics and Q-CHAT scores.

Q-CHAT	TBV	Sex	Age at Q-CHAT	Maternal age	Birth Weight	Age at scan
dHCP, n = 454	***r*** = **−0.14**	***r*** = **0.12**	*r* = −0.04	***r*** = **−0.11**	*r* = 0.01	r = 0.02
	***p*** = **0.003**	***p*** = **0.009**	*p* = 0.379	***p*** = **0.019**	*p* = 0.866	p = 0.642
model adjusted R^2^ = 0.05, p < 0.00001						
CHILD, n = 24	***r*** = **−0.51**	*r* = 0.06	*r* = −0.10	*r* = −0.19	*r* = 0.35	*r* = −0.03
	***p*** = **0.030**	*p* = 0.818	*p* = 0.693	*p* = 0.441	*p* = 0.152	*p* = 0.893
model adjusted R^2^ = 0.20, p = 0.14						
**Q-CHAT**	***Grey Matter***	**Sex**	**Age at Q-CHAT**	**Maternal age**	**Birth Weight**	**Age at scan**
dHCP, n = 454	***r*** = **−0.11**	***r*** = **0.11**	*r* = −0.05	***r*** = **−0.11**	*r* = −0.01	r = 0.02
	***p*** = **0.019**	***p*** = **0.020**	*p* = 0.260	***p*** = **0.014**	*p* = 0.913	p = 0.634
model adjusted R^2^ = 0.05, p = 0.0002						
CHILD, n = 24	***r*** = **−0.51**	*r* = 0.06	*r* = −0.10	*r* = −0.19	*r* = 0.35	*r* = −0.03
	***p*** = **0.032**	*p* = 0.801	*p* = 0.706	*p* = 0.461	*p* = 0.156	*p* = 0.893
model adjusted R^2^ = 0.20, p = 0.15						
**Q-CHAT**	**White Matter**	**Sex**	**Age at Q-CHAT**	**Maternal age**	**Birth Weight**	**Age at scan**
dHCP, n = 454	***r*** = **−0.15**	***r*** = **0.13**	*r* = −0.04	***r*** = **−0.11**	*r* = 0.01	r = −0.04
	***p*** = **0.002**	***p*** = **0.006**	*p* = 0.346	***p*** = **0.021**	*p* = 0.884	p = 0.351
model adjusted R^2^ = 0.06, p < 0.00001						
CHILD, n = 24	***r*** = **−0.50**	*r* = 0.02	*r* = −0.12	*r* = −0.22	*r* = 0.38	*r* = −0.07
	***p*** = **0.034**	*p* = 0.947	*p* = 0.626	*p* = 0.373	*p* = 0.123	*p* = 0.790
model adjusted R^2^ = 0.19, p = 0.15						
**Q-CHAT**	**CSF**	**Sex**	**Age at Q-CHAT**	**Maternal age**	**Birth Weight**	**Age at scan**
dHCP, n = 454	*r* = −0.09	*r* = 0.09	*r* = −0.06	***r*** = **−0.12**	*r* = −0.01	r = −0.06
	*p* = 0.070	*p* = 0.063	*p* = 0.205	***p*** = **0.010**	*p* = 0.815	p = 0.233

r is the partial coefficient of each variable. p is uncorrected value of significance in multiple regression. Bold font indicates uncorrected significance at p < 0.05.

*TBV* total brain volume.

model adjusted R^2^ = 0.04, p < 0.0004.

### Postnatal global brain metrics and autistic traits in the CHILD cohort

Consistent findings were found in the CHILD cohort (n = 27), in which infants were scanned at a later time-point during infancy (mean scan age = 50.45, SD = 2.8 weeks post-conception). Total brain volume at this point, as well as total grey matter and white matter, were negatively associated with autistic traits on the Q-CHAT, after controlling for the same covariates as the linear regression models above (Fig. 3B)(Table 2). Post-hoc sensitivity analysis showed that this effect was driven by individuals with a reported first degree relative with diagnosed autism in this cohort (n = 7, interaction term partial r = 0.61, p = 0.01) (Fig. 3C).

### Postnatal regional brain volumes and autistic traits in the dHCP cohort

To test for potential regional differences, association analyses were conducted in the dHCP cohort for the volume of all available brain segments. This included 82 cortical, subcortical grey and white matter segments in the infant brain, based on the published structural pipeline data of the dHCP and excluding CSF, background measures and ventricles [19, 20]. The volumes in these grey and white matter segments were tested for an association with Q-CHAT scores of the children at 18 months (n = 454, mean Q-CHAT score = 30.14 (SD = 8.3) after exclusion criteria applied) using two main statistical models. Model 1 was controlled for total brain volume, to test for regional volume effects on Q-CHAT scores that were independent to the total brain effect identified above. Model 2 included an interaction term of total brain volume with the regional volume for each segment. Correction for multiple testing was done via application of the Benjamini-Hochsberg false discovery rate (FDR) (n = 82 tests) [21].

At an uncorrected level of significance (p < 0.05), several regions were associated with Q-CHAT scores but none of these results remained significant following correction via FDR for 82 tests. The patterns of non-significant results at uncorrected p < 0.05 differed for each statistical Model (Table 3 for Model 1, Supplementary Table 3 for Model 2).

**Table 3:** All regions in the dHCP that showed non-significant trends toward association with later Q-CHAT scores when controlling for total brain volume (TBV), at uncorrected p < 0.05.

	dHCP Model 1	Regional volume	Total Brain Volume	Sex	Maternal age
GM	Posterior parahippocampal gyrus (L)	r = 0.114	r = −0.180	r = 0.137	r = −0.115
		p = 0.016	p < 0.001	p = 0.004	p = 0.015
WM	Posterior medial-inferior temporal gyri (R)	r = −0.146	r = 0.003	r = 0.142	r = −0.107
		p = 0.002	p = 0.951	p = 0.003	p = 0.023
	Posterior parahippocampal gyrus (R)	r = 0.145	r = −0.196	r = 0.134	r = −0.118
		p = 0.002	p = 0.000	p = 0.005	p = 0.012
	Frontal lobe (R)	r = −0.096	r = 0.032	r = 0.130	r = −0.107
		p = 0.043	p = 0.497	p = 0.006	p = 0.023
	Thalamus low intensity part (L)	r = 0.093	r = −0.163	r = 0.123	r = −0.111
		p = 0.049	p = 0.001	p = 0.009	p = 0.019

Model included these covariates, as well as age at Q-CHAT measurement and birth weight (data not shown, all non-significant).

r is the partial coefficient of each variable. p is uncorrected value of significance in multiple regression.

*GM* grey matter, *WM* white matter.

**Fig. 4:**
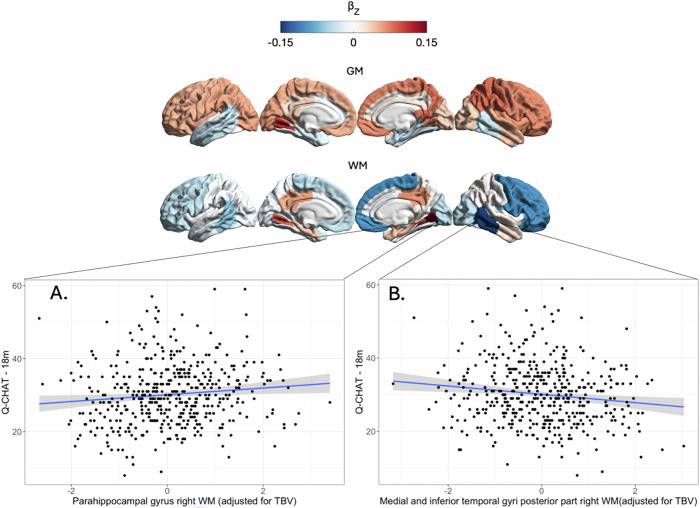
Regional patterns of association with traits. Atlas showing the effect sizeof association with Q-CHAT scores(Model 1) for each regional grey matter(GM) and white matter (WM) segments, regardless of statistical significance. Following correction for multiple testing, two white matter segments showed a non-significant trend towards association (FDR-q < 0.1, corresponding to p < 0.005): (**A**) Parahippocampal gyrus (Left – WM) showed a positive non-significant trend and (**B**) medial-inferior temporal gyrus (Right- WM) showed a negative non-significant trend of association with Q-CHAT scores after controlling for TBV (values shown in graphs have been adjusted for TBV, to illustrate direction of effect in Model 1).

After correction with FDR, Model 1 identified two white matter segments in the right posterior temporal lobe that showed a non-significant trend towards association with Q-CHAT scores (FDR-q < 0.1, p < 0.005). The direction of this non-significant trend differed, with volume in the posterior region of the medial and inferior temporal gyri showing a non-significant trend towards negative association, and volume in the parahippocampal gyrus showing a non-significant trend towards a positive association with Q-CHAT scores, after controlling for the negative effect of total brain volume and other variables (Model 1)(Fig. 4).

Model 2 did not identify any white matter or grey matter regions that significantly interacted with total brain volume in their association with Q-CHAT scores or showed a similar non-significant trend towards an association (FDR-q < 0.1, p < 0.005)(Supplementary Table 3, Supplementary Fig. 3).

### Postnatal regional brain volumes and autistic traits in the CHILD cohort

Results in the dHCP were used to inform a region-of-interest (ROI) analysis of cortical segments in the independent CHILD cohort (n = 27 scanned infants at mean age = 50 (SD = 2.9) weeks post-conception). This ROI analysis of n = 15 regions included several regions in the frontal lobe, temporal lobe, as well as the anterior cingulate, thalamus and subthalamic nuclei, matching the regions in which non-significant results were found in the dHCP cohort (Supplementary Table 4).

**Fig. 5:**
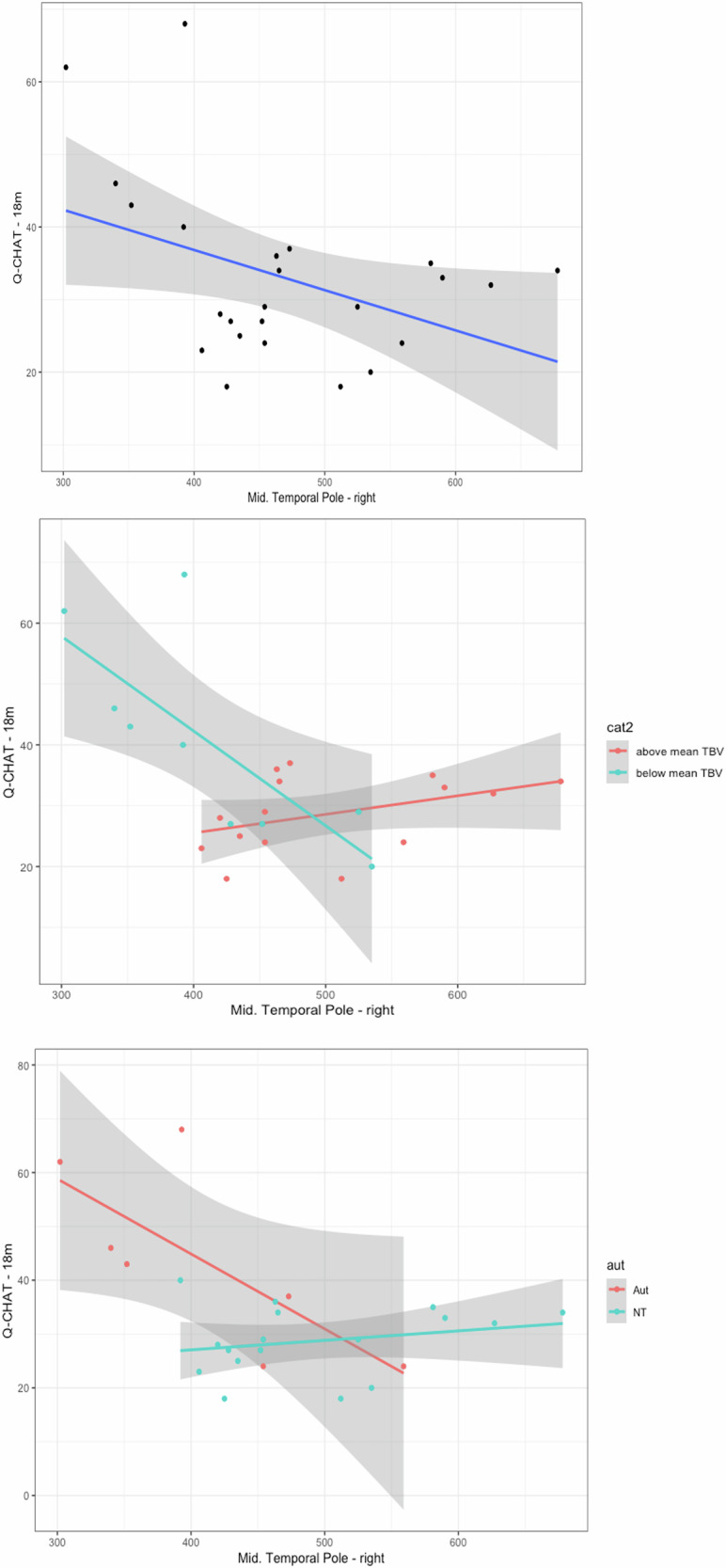
ROI analysis in the CHILD cohort. **A** - **Top graph**. In the CHILD cohort, volume in the right middle temporal gyrus is associated negatively with Q-CHAT scores, **B - Middle graph**. with a non-significant trend for an interaction with TBV (total brain volume)(FDR-q < 0.1), **C - Bottom graph**. and this effect is driven by individuals with family history of autism (‘Aut’).

In this ROI analysis in the CHILD cohort, Model 1 did not identify any regional associations with Q-CHAT scores that were independent to the negative effect of total brain volume (Supplementary Table 5). Model 2 showed that the volumes of the right temporal pole, middle and inferior temporal gyri were significantly associated with Q-CHAT scores (FDR-adjusted q < 0.05) (Table 4)(Fig. 5A), with a non-significant trend towards an interaction with total brain volume (p < 0.05, FDR-adjusted q = 0.07) (Table 5). The positive effect size of the interaction term indicated a non-significant trend for opposing effects on Q-CHAT, between total brain volume and these regional volumes in the temporal lobe (Fig. 5B). Further post-hoc analysis showed that these results were driven by the participants with a diagnosed relative with the condition (n = 7) (Fig. 5C).

**Table 4:** All regions tested in the CHILD cohort in an ROI analysis of the volume of n = 15 segments.

CHILD Model 2	Regional volume	TBV	Regional x TBV	Sex	Maternal age
Temporal_Pole_Mid_R	r = −0.671*	r = −0.714	r = 0.659	r = 0.153	r = 0.118
	p = 0.004*	p = 0.002*	p = 0.006	p = 0.571	p = 0.662
Temporal_Mid_R	r = −0.614*	r = −0.624	r = 0.592	r = −0.058	r = 0.073
	p = 0.011*	p = 0.010	p = 0.016	p = 0.831	p = 0.788
Temporal_Inf_R	r = −0.607*	r = −0.606	r = 0.585	r = 0.024	r = 0.148
	p = 0.013*	p = 0.013	p = 0.017	p = 0.931	p = 0.584
Temporal_Pole_Sup_R	r = −0.622*	r = −0.600	r = 0.574	r = −0.121	r = 0.095
	p = 0.010*	p = 0.014	p = 0.020	p = 0.655	p = 0.726
Amygdala_R	r = −0.504	r = −0.617	r = 0.543	r = −0.042	r = 0.009
	p = 0.047	p = 0.011	p = 0.030	p = 0.877	p = 0.973
ParaHippocampal_L	r = −0.424	r = −0.520	r = 0.450	r = −0.032	r = −0.154
	p = 0.102	p = 0.039	p = 0.080	p = 0.906	p = 0.569
Subthalamic_Nuc_R	r = −0.443	r = −0.491	r = 0.436	r = 0.010	r = −0.095
	p = 0.086	p = 0.053	p = 0.092	p = 0.971	p = 0.727
Frontal_Mid_R	r = −0.272	r = −0.454	r = 0.324	r = −0.038	r = −0.157
	p = 0.309	p = 0.078	p = 0.221	p = 0.889	p = 0.560
ParaHippocampal_R	r = −0.316	r = −0.356	r = 0.320	r = −0.054	r = −0.055
	p = 0.233	p = 0.175	p = 0.228	p = 0.843	p = 0.841
Cingulum_Ant_R	r = −0.147	r = −0.432	r = 0.233	r = −0.088	r = −0.069
	p = 0.587	p = 0.095	p = 0.386	p = 0.745	p = 0.801
Subthalamic_Nuc_L	r = −0.241	r = −0.309	r = 0.229	r = −0.095	r = −0.051
	p = 0.369	p = 0.243	p = 0.393	p = 0.725	p = 0.850
Thalamus_R	r = −0.299	r = −0.247	r = 0.227	r = −0.022	r = −0.091
	p = 0.261	p = 0.356	p = 0.399	p = 0.934	p = 0.738
Fusiform_L	r = −0.169	r = −0.307	r = 0.198	r = −0.051	r = −0.135
	p = 0.531	p = 0.248	p = 0.462	p = 0.853	p = 0.619
Thalamus_L	r = −0.215	r = −0.232	r = 0.196	r = −0.027	r = −0.112
	p = 0.425	p = 0.387	p = 0.468	p = 0.921	p = 0.679
Frontal_Sup_R	r = −0.189	r = −0.002	r = 0.018	r = −0.111	r = −0.201
	p = 0.484	p = 0.993	p = 0.946	p = 0.682	p = 0.456

Data shown for Model 2, which included interaction term with total brain volume (TBV), the covariates shown, as well as birth weight and age at Q-CHAT (not shown, all non-significant).

Asterisk denotes significance at FDR-q < 0.05.

**Table 5:** Brain regions in the dHCP with non-significant trends of association between prenatal volume and later Q-CHAT after controlling for total brain volume (TBV).

	dHCP Model 1	Regional volume	TBV	Sex	Maternal age
GM	Brainstem (midline)	r = −0.380	r = −0.077	r = 0.248	r = 0.105
		p = 0.003	p = 0.562	p = 0.058	p = 0.430
	Lateral occipitotemporal & anterior fusiformis gyrus (L)	r = 0.349	r = −0.376	r = 0.219	r = 0.077
		p = 0.007	p = 0.003	p = 0.095	p = 0.564
	Lateral occipitotemporal & anterior fusiformis gyrus (R)	r = 0.258	r = −0.291	r = 0.222	r = 0.051
		p = 0.048	p = 0.025	p = 0.095	p = 0.704
WM	Posterior Cingulate Gyrus (L – WM)	r = 0.324	r = −0.371	r = 0.220	r = 0.052
		p = 0.012	p = 0.004	p = 0.095	p = 0.697

Model included these covariates, as well as age at Q-CHAT measurement and birth weight (data not shown, all non-significant).

r is the partial coefficient of each variable. p is uncorrected value of significance in multiple regression.

*GM* grey matter, *WM* white matter.

### Fetal brain volume and autistic traits in the dHCP

To investigate fetal brain volume and avoid repeat testing of the same participants for the same outcome, a separate cohort was created in the dHCP of individuals that had only underwent fetal volumetric measurements and were not included in the prior analyses of infant brain volume (Fig. 1). If a participant was scanned more than once prenatally (n = 12, after exclusion criteria applied), the latest fetal scan was chosen, to reduce the total variance in gestational age at scan of the cohort (final mean fetal age at scan = 204.6(SD = 26.3) days post-conception). In total, n = 106 of these scans could be paired to a Q-CHAT score.

In this subset, no associations were found between autistic traits on the Q-CHAT and fetal total brain volume, or other global metrics (Supplementary Table 6). Analysis of all the regional volumes of grey and white matter segments, using the same two linear regression models described above, yielded only non-significant findings at uncorrected p < 0.05 (Table 5), which were not significant after correction for multiple testing.

Further testing of equivalent fetal brain segments in the CHILD cohort (Supplementary Table 4) did not yield findings at either uncorrected or corrected level of significance.

### Perinatal brain growth and autistic traits

**Fig. 6:**
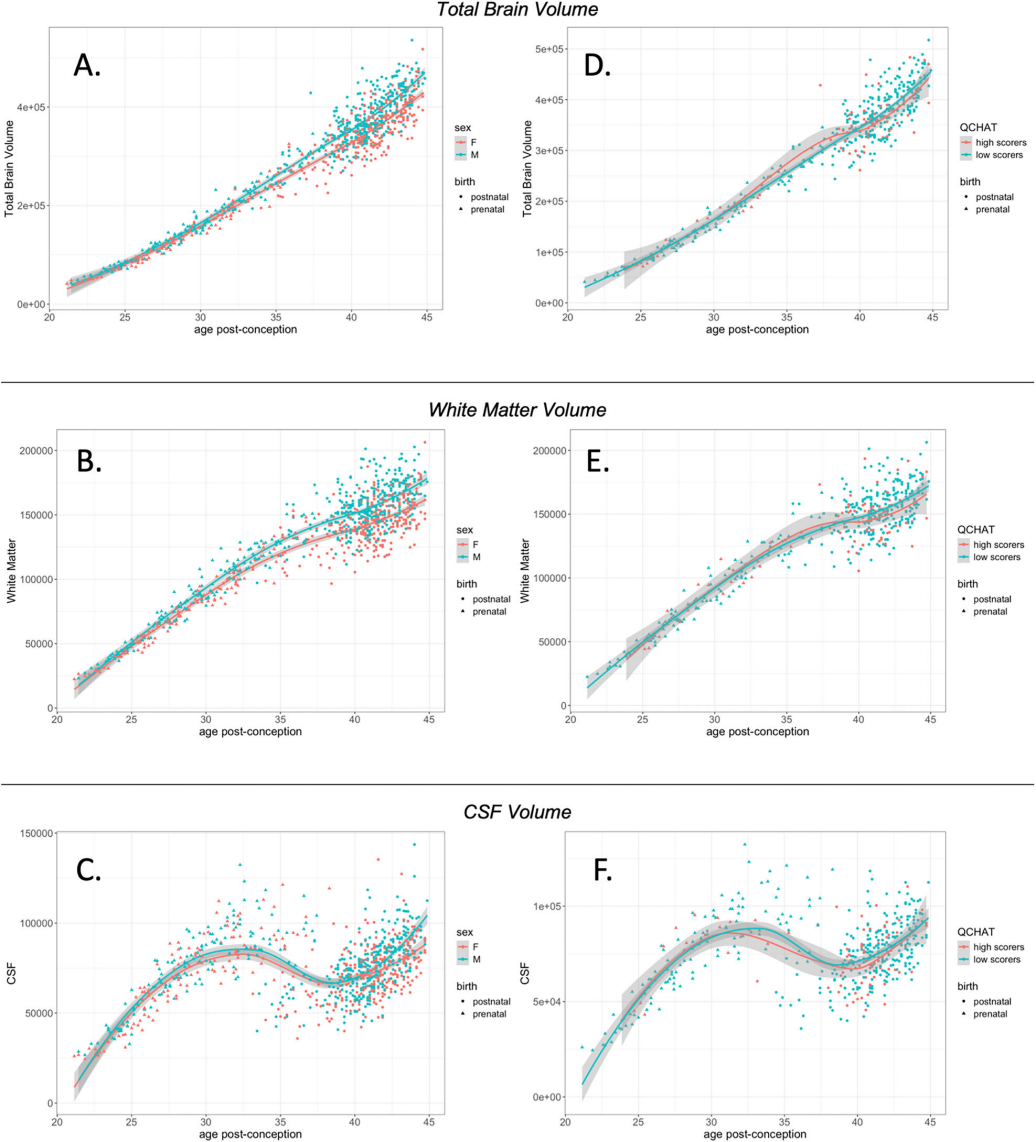
Perinatal brain growth and autistic traits. In the dHCP, brain volume of both prenatal and postnatal scans based on age post-conception for (**A**): total brain volume, (**B**) white matter volume and (**C**) CSF volume. (**D,** **E,** **F**): Same data with separate curves fitted for Q-CHAT high scorers (> = 39) and low-scorers ( < 21) (participants in the typical range omitted in plots D to F).

To investigate perinatal total brain growth over the perinatal period, infant volumetric data was combined with fetal data from the dHCP, generating a cohort of participants who were scanned both prenatally and postnatally (n = 79). To study the association of total brain growth rates with autistic traits as the dependent variable, a new variable was calculated to capture the change rate of each total brain metric, standardised for age post-conception (Eq. 1 in Methods). Rates of change for total brain volume, cortical grey matter and white matter were positive and higher in males compared to females. The rate of CSF change was more variable, with many individuals in the dHCP showing a reduction of CSF volume between the prenatal and postnatal scan (Fig. 6C).

No association was found between the rate of total brain volume, grey matter or white matter change, and later Q-CHAT scores in linear regression models that also controlled for sex, maternal age, age at the time of Q-CHAT measurement (adjusted for gestational age at birth) and birth weight. The rate of CSF change was significantly and positively associated with Q-CHAT scores after controlling for the same covariates (n = 79, partial-b = 0.35, p = 0.002) (Fig. 6F). This effect was driven by the individuals with a postnatal reduction in CSF, who, in turn, were more likely to have lower autistic traits (Supplementary Fig. 4F). Post-hoc sensitivity analyses showed that this effect remained statistically significant when restricting the analysis to infants born without C-section, or when outliers with very high perinatal CSF reduction (n = 1) or any premature births (n = 2) were excluded.

This apparent perinatal reduction in CSF for a subset of individuals was not found in the CHILD cohort, in which infants all had postnatal increases in CSF volume, when scanned at a later-point of infant development (mean scan age = 50.45, SD = 2.8 weeks post-conception), compared to the dHCP(mean age = 41.28 [SD = 1.93] weeks post-conception). The rate of CSF change in CHILD was not significantly associated to later Q-CHAT scores (n = 21, partial - b = −0.37, p = 0.09) (Supplementary Fig. 5).

## Discussion

This is the first study of early neurodevelopment that includes both prenatal and postnatal brain scans, as well as perinatal brain growth rates, with an aim to examine their association to the emergence of the first autistic traits at 18 months of age. In summary, this study did not find evidence that is consistent with the ‘brain overgrowth’ hypothesis of autism, nor evidence of a sex-mediated interaction in this regard. On the contrary, total brain volume, grey matter and white matter in early infancy were all negatively associated with autistic traits (Q-CHAT at 18 months) in individuals in the developing Human Connectome Project (dHCP), who were scanned soon after birth. This was also found when assessing brain structure at a slightly later time-point, in an independent, smaller cohort in Cambridge, where infants were scanned by the second month of life as part of the CHILD study (mean scan age = 50.45, SD = 2.8 weeks post-conception). Association analyses that focused on specific brain segments, in both cohorts, indicated significant heterogeneity between the datasets, with some regions, particularly in the temporal lobes warranting further discussion.

Previous neuroimaging studies of autistic traits in childhood and in adulthood have also reported negative associations with multiple regional brain volumes [8, 10], as well as with various total brain measures. A recent longitudinal MRI study of autistic individuals also did not find evidence consistent with “brain overgrowth” during childhood [22]. Besides brain imaging via MRI, a studies that assessed head circumference prenatally also reported mixed findings, with both smaller and larger differences, for autistic traits in childhood, as well as for individuals with clinically diagnosed autism [17, 18]. Clinical cases of microcephaly, macrocephaly/hydrocephalus, as well as genetic mutations relating to both, are all enriched in neonates that are later diagnosed with autism [23, 24].

This heterogeneity could be attributed to significant population-level variance, as well as differences in the underlying neurostructural substrates of autistic traits themselves. Different autism sub-types may differ substantially in their developmental trajectories, depending on their genetic profile, chromosomal sex and perinatal clinical context. In the wider clinical context, in the field of metabolic health, neonates affected by suboptimal pregnancy conditions are often characterised by general signs of undergrowth (e.g., low birth weight) and are often reported to show evidence of pronounced ‘catch-up’ in later life, which may go over and above their unaffected peers and lead to maladaptive ‘excess’ (e.g., metabolic syndrome) [25, 26]. This is consistent with the finding in one of the earliest clinical studies of autistic children’s brain growth, in which autistic children were found to have lower head circumference at birth, which was then followed by apparent brain ‘overgrowth’ in later infancy [15]. Unfortunately, both the dHCP and CHILD cohorts did not re-scan their participants in later infancy or toddlerhood, preventing the study of a potential ‘catch-up overgrowth’ effect in later postnatal life.

After controlling for total brain volume, the association of grey matter volume with Q-CHAT did not yield statistically significant results, but a pattern was noted showing positive values for frontal areas, but negative values for temporal areas (Fig. 4). This is broadly consistent with the findings of a recent study in a subset of the dHCP cohort (n = 273 term neonates), which reported associations between polygenic scores (PRS) of autism and the volume of various brain lobes at birth [27]. After controlling for total brain volume, these associations were positive for fronto-termporal areas, and negative for parieto-occipital areas (including the fusiform gyrus), indicating that the anterior-posterior axis of cortical patterning may be a relevant area for future research.

With regards to white matter, in this study, after controlling for total brain volume, two regions of the right posterior temporal lobe showed noteworthy patterns at an uncorrected level of significance. These results were not statistically significant after controlling for multiple testing (n = 82 tests), albeit a non-significant trend was noted (FDR-q < 0.1). Interestingly, the direction of effect was different between white matter in the parahippocampal gyrus and a segment of the outer temporal lobe that included parts of the medial and inferior temporal gyrus. Despite their relative proximity, the former showed a positive effect size for Q-CHAT, while the latter showed a negative effect size, which were independent to the significant negative effect of total brain volume.

Early fMRI studies have linked the parahippocampal region to visuospatial processing (e.g. in object-background processing) [28], contextual associations of objects [29], as well as in determining the emotional salience of audiovisual stimuli (e.g. of happy/sad music) [30]. Lesion studies are also consistent with these effects [31, 32], while direct stimulation of the area has been shown to lead to visuospatial hallucinations [33]. In infants, more recent functional studies have shown that the specificity of the parahippocampal area to the processing of objects and ‘scenes’ (rather than faces) may be present from a very young age ( < 9 months) [34, 35]. On the other hand, the findings regarding the temporal lobe are consistent with previous findings on autistic traits [8] or on diagnosed autism [36–39] and indicate the neurodevelopmental significance of areas that have been linked to speech, language, and face processing, as indicated by functional studies in infants and lesion studies in adults [40, 41].

The temporal lobe and especially grey matter in the lateral occipital-temporal and fusiform gyrus, was also indicated in the analysis of fetal brain volume in the dHCP. In both hemispheres, fetal brain volume in these regions showed non-significant trends for positive associations with later autistic traits, at an uncorrected level of significance. However, this effect was not found for equivalent regions in CHILD or for postnatal grey matter in the dHCP cohort. This may be due to methodological differences between the two cohorts, underlying heterogeneity or a potential ‘catch-up’ effect that may allow for more variance prenatally rather than postnatally for grey matter.

It is important to note that these regional differences represent volumetric associations, which characterise heterogeneous cohorts and do not necessarily correspond to specific functionalities. However, the emerging patterns could help inform further research into the neural substrates of autistic traits. In particular, the contrasting non-significant patterns found for postnatal white matter volumes in the dHCP, could be understood to parallel the empathising/systemising profiles that have been proposed to characterise autism and autistic traits, based on clinical, behavioural observations and self-report questionnaire data over the years [42, 43]. Future research could explore if similar patterns in temporal lobe connectivity are found in later ages, as well as the way white matter growth in the temporal lobes may interact with total brain volume growth.

Finally, it is important to highlight that while most global brain metrics appeared to increase between the prenatal and postnatal measurements, CSF appeared to decrease transiently after birth in some individuals of the dHCP cohort, as has been reported before [44]. This is consistent with some clinical reports that describe a decline in CSF in the first week of life, possibly due to the process of labour and the transient transfer of CSF from the cranium to the spinal duct [45]. Interestingly, individuals that scored high on the Q-CHAT appeared to have a smaller perinatal reduction in their CSF. This is consistent with previous findings in other postnatal cohorts of autistic individuals scanned later in infancy (6 months onwards) which found higher CSF volume [46, 47], although it remains unclear if this can be attributed to factors such as the mode of delivery or duration of labour. To gain insight into this finding and the noted interactions between total brain and regional volumes, normative modelling and better powered longitudinal data are required.

This study is also limited by the differences between the two cohorts, which included different segmentation methods of the infant and fetal brain [48], different magnetic field intensities and slightly different time-points of postnatal scanning, with measurements in the dHCP taking place closer to birth. The CHILD cohort is particularly small and any results should be viewed as exploratory trends that warrant further investigation. In addition, the behavioural patterns that are measured by the Q-CHAT rely on parent-report and may be capturing differences in the rate of neurodevelopmental and socio-cognitive milestones, rather than a specific precursor to clinical autism. The statistical models that showed total brain undergrowth in infants with high autistic traits, were all controlled for birth weight, adjusted for age at birth and did not include very or extremely preterm children, who may have confounded the association. In addition, the screening potential of the Q-CHAT for autism, as well as the observed small sex difference in scores, have both been confirmed in independent cohorts around the world [4–6]. Furthermore, in the CHILD cohort, family history of autism appeared to have an additive effect in the negative association with total brain volume. Future research could expand on these interactions, by considering other perinatal factors that may cause neurodevelopmental differences, as well as include polygenic scores for conditions, such as autism or ADHD, to determine specificity.

In conclusion, this study finds evidence of reduced brain volume soon after birth in individuals with high autistic traits in the second year of life. This effect appears to affect grey matter as well as white matter bilaterally and to become apparent soon after birth rather than prenatally. This global brain effect may interact with regional differences, particularly within the temporal lobe, as well as with family history for autism. Further research is required, regarding postnatal brain growth trajectories, sex differences and functional connectivity patterns that are specific to early cognition and autism, particularly in the temporal lobes.

## Methods

### The developing human connectome project (dHCP)

The dHCP is an open-science dataset containing multimodal measures of perinatal brain development and future outcomes, including both fetal and infant scans, with open-access protocols [49, 50]. Data were provided by the developing Human Connectome Project, KCL-Imperial-Oxford Consortium funded by the European Research Council under the European Union Seventh Framework Programme (FP/2007-2013) / ERC (European Research Council) Grant Agreement no. [319456].

Recruitment and obtaining of informed consent took place at St. Thomas’s Hospital (London, UK) and imaging at the Evelina Newborn Imaging Centre, Centre for the Developing Brain, King’s College London. The infant dataset contains 783 individuals, of which 583 were born at term equivalent age without any pregnancy or neonatal problems, as reviewed by neuroradiologists. The fetal dataset contains 273 individuals (GA: 21-38 weeks).

### MRI acquisition - dHCP

Infant participants were imaged during natural sleep. Six were sedated with chloral hydrate. All fetal and infant scans were motion corrected with additional details available in published reports [51–53]. Infants were imaged with auditory protection after feeding. In the case of both fetuses and infants, scanning was completed using a 3 T Philips Achieva scanner. 2D snapshot multi-slice acquisitions were used to freeze fetal movements, while infant scans utilized a 32 channel phased array head coil. Pulse oximetry, respiration and body temperature of infants were monitored throughout the session. The imaging cradle system placed infants in a standardized pose with a field of view that accommodated 95% of late-term neonates. The scanner software ramped up gradient waveforms over 5 s prior to data acquisition or radiofrequency pulses to avoid waking the infants. Calibration scans and anatomical images were acquired at 27 slices/second.

### Structural scans - dHCP

Imaging parameters were optimized for contrast to noise ratio. For infant scans, the nominal relaxation parameters for grey matter T1/T2 images were 1800/150 ms and white matter T1/T2: 2500/250 ms (Williams et al., 2005). T2w multi-slice FSE images were acquired in sagittal and axial slice stacks. The parameters were as follows: in-plane resolution of 0.8 ×0.8 mm2, 1.5 mm slices overlapped by 0.8 mm, −22w: TR/TE = 12000/156 ms, SENSE factor 2.11 (axial) and 2.60 (sagittal). 3D MPRAGE images were acquired with 0.8 mm isotropic resolution and parameters (TR/TI/TE = 11/1400/4.6 ms; SENSE factor 1.2 RL). For the fetal scans, the mother was placed in a supine position and images were acquired using a 32-channel cardiac coil. Blood pressure and peripheral pulse oximetry were monitored throughout the scan. Structural T2w data were acquired from 6 uniquely oriented stacks centred to the fetal brain using a zoomed multi-band 2 (MB2) single-shot Fast Spin Echo (ssFSE) sequence. The parameters were as follows: in plane resolution 1.1×1.1×2.2 mm (−1.1 mm gap), TR/TE = 2265/250 ms.

Structural MRI data obtained postnatally and prenatally were processed separately based on the respective published structural pipelines [19]. These utilised a series of segmentation methods for broad tissue categories (n = 9 for both infant and fetal scans), as well as detailed grey and white matter measurements (n = 87 for infant and n = 91 for fetal scans). All fetal segmentations were reviewed for anatomical accuracy. Mislabelling errors were manually corrected by a neuroscientist with expertise in fetal neuroanatomy. Preprocessing and structural acquisition codes are all available online.

### The cambridge human infant and longitudinal development (‘CHILD’) study

Pregnant women were recruited to take part in CHILD at the Rosie Maternity Hospital, in Cambridge, via advertising material or in-person discussions in the prenatal ultrasound unit, during their first or second pregnancy monitoring appointment. An additional subset of the cohort consisted of pregnant women with a family history of autism, having themselves, their partner, and/or a previous child received an autism diagnosis. These “high likelihood” participants were recruited through the Cambridge Autism Research Database, support groups across the UK, social media that are specific to the condition, and adverts placed on magazines. Exclusion criteria for mothers included multiple births, the consumption of alcohol, and smoking (determined in an initial screening form).

Participants agreed to take part in two MRI brain scans of their child’s brain, prenatally during 30–33 weeks of pregnancy (fetal stage) and postnatally at 8–12 weeks after birth (infant stage). Infant scans were conducted during natural sleep with no sedation used.

Two typical-likelihood infants and one high-autism-likelihood infant could not be followed-up for the infant scan. Scanning was therefore conducted with 40 participants at the postnatal stage. Structural data was not obtained from one autism high-likelihood participant, due to movement artefacts. Moreover, data was not obtained from 11 participants (of whom one was in the high-likelihood group) due to the infant waking and showing distress in the MRI scanner or due to the inability of the infant to achieve sleep. Therefore, at the infant stage, structural MRI scans were available for 27 participants (n = 13 female), of which 7 were at high familial likelihood for autism (n = 3 female). None of the infants included in the study during the infant stage had been born preterm ( < 37 weeks gestation at birth).

### MRI Acquisition - CHILD

All magnetic resonance images were obtained at the Paediatric Scanning Unit of the Rosie Hospital, Cambridge, UK with a 1.5 Tesla GE scanner. Structural images were acquired using the balanced steady-state gradient echo sequence FIESTA (Fast Imaging Employing Steady-state Acquisition). The following parameters were used: repetition time of 3.5 ms, echo time of 1.4 ms, a flip angle of 55°, 172 slices with a slice thickness of 1 mm, voxel size of 1.875 ×1.875 x 1mm3, field of view of 256 ×256.

### Structural image pre-processing - CHILD

Fetal brain images were manually reoriented using the Reorient tool in ITK-SNAP [54] as *in utero* head movement led to mismatches between image orientation codes and the actual orientation of the fetal brains. This step was not necessary for infant structural images as infants were scanned during natural sleep with cushion padding either side to minimise movement within the scanner. Spatial origin was set to the Anterior Commissure fibre tract permitting accurate co-registration of reoriented images. All images were then manually skull stripped with the resulting ROI extending to the skull/CSF interface and to the base of the Cerebellum. Using a sex balanced random sample of individuals with both fetal and infant images a study specific structural template was generated using Advanced Normalisation Tools (ANTs) [55].

The STA31 template, part of a spatiotemporal MRI atlas for segmentation of early brain development [56] together with all fetal and infant structural images were registered to the study specific template using ANTs. So as not to introduce interpolation artefacts, STA31-specific-template and individual-specific-template spatial warps were combined into a single inversely symmetrical STA31-to-individual transformation. As it was not possible to directly segment brain images into grey and white matter, due to insufficient grey/white matter contrast during this developmental period, STA31 parcellations were re-sampled into subject space to indirectly assess grey matter properties within the brain. The sum of combined re-sampled STA31 segmentations and parcellations was then used to estimate total brain volume from the structural images.

### Autistic traits

Autistic traits were measured using the Quantitative Checklist for Autism in Toddlers (Q-CHAT). Q-CHAT is a 25-item questionnaire assessing autistic traits in children ages 18–36 months of age. A score of 39 has been proposed as a cut-off of clinical significance for screening and further neurodevelopmental assessment [6]. For the dHCP, 619 (79%) infants attended a follow-up assessment for various behavioural questionnaires and anatomical measurements, during which parents were asked to fill in the Q-CHAT. For the CHILD, following informed consent both at the point of recruitment and before their child’s postnatal brain scan via MRI, participating mothers were invited via email to complete a series of online questionnaires on their child’s development, including the Q-CHAT, 18 months after the birth of their child, on the online platform Qualtrics. Anonymised data was then copied onto RedCap; an online platform for secure storage and data analysis, used by research staff and students at the University of Cambridge [57].

### Statistical analyses

In the dHCP cohort, infant total brain metrics included for analyses included total grey matter, total white matter, CSF (excluding ventricles), and total brain volume (TBV), which in turn was calculated based on the results of the study’s structural pipeline (either infant or fetal) and specifically the total sum of all regions included in the ‘DrawEM-9’ segmentation of the brain in 9 ‘tissues’, namely white matter, grey matter, deep brain structures, brainstem, and cerebellum, excluding volumetrics measures of CSF and ventricles. For fetal total brain metrics, the ‘DrawEM-17’ segmentation was used instead, with total brain volume derived from the sum of all segments, excluding CSF, both lateral ventricles, the third and fourth ventricle, as well as the cavum septum pellucidum (CSP).

Prior to any statistical testing or association models, all postnatal ages were adjusted to reflect age post-conception, by adding individuals’ gestational age at birth to their reported postnatal age. In the CHILD cohort, where gestational age at birth was missing from study records (n = 3), it was assumed that the neonate had achieved term gestation (40 weeks).

### Association analyses with Q-CHAT scores - dHCP

Total brain metrics (total brain volume, cortical grey matter, total white matter, and CSF) were assessed for association to Q-CHAT scores using separate linear regression models that were controlled for age at scan, age at Q-CHAT report, sex, maternal age, and birth weight. Postnatal ages were combined with gestational weeks to birth to reflect age post-conception. The statistical models were conducted separately for postnatal scans and prenatal scans in non-overlapping cohorts of the dHCP.

In addition, exploratory regional analyses were conducted using all available segments of the infant and fetal structural pipelines, excluding CSF, ventricles, the cavum septum pellucidum and background measurements. Two linear regression models were used (‘Model 1’ and ‘Model 2’), controlling for the same covariates as the association analyses of total brain metrics. In addition to these, Model 1 also controlled for total brain volume, while Model 2 also included an interaction term between regional volume and total brain volume. The aims of these models were to explore regional effects that were independent of the main or interaction effect of total brain volume, respectively, in their association to Q-CHAT scores. Noteworthy “trends” for an association were considered if uncorrected significance was at 5%, while statistical significance was decided based on a level of 5% following correction based on the false discovery rate (FDR) that was calculated for comparisons involving white matter and grey matter segments (including subcortical structures and the cerebellum, total number of tests n = 82).

Finally, fetal and infant measurements were combined where available for the same participant to examine the association between the rate of change of total brain metrics and Q-CHAT scores. The rate was calculated based on the following equation:

1 $$\begin{array}{c}(Total \, Brain \, metric \, at \, infant \, scan - Total \, Brain \, metric \, at \, fetal \, scan)\\ /\\ (age \, post{\hbox{-}}conception \, at \, postnatal \, scanning-age \, post{\hbox{-}}conception \, at \, prenatal \, scanning)\end{array}$$

This was done in order to standardise for the wide range in scanning ages, and test for an association, while maintaining Q-CHAT scores as the dependent variable in question.

The association of the rate of change for each total brain metrics was then tested for association with Q-CHAT scores in linear regression models that also controlled for sex, maternal age, birth weight and age at the time of Q-CHAT report.

Specifically for illustrative purposes, a categorical variable with three levels for Q-CHAT was created based on the following thresholds derived from the cohort’s mean and range of two standard deviations: ‘low-scorers’: Q-CHAT score<21, ‘high scorers’: Q-CHAT score > = 39, ‘typical range’: Q-CHAT score: between 21 and 38. This approach was based on the properties of the Q-CHAT distribution in the dHCP cohort, as well as previous suggestions of 39, as the threshold of “clinical significance” that warrants closer monitoring and a subsequent diagnostic assessment [3, 4].

A categorical variable based on TBV distribution postnatally was also created with the following cut-offs: “micro”: (mean of TBV – standard deviation), “macro” : (mean of TBV + standard deviation), “typical”: range of 2 standard deviations around mean.

The assumptions of all highlighted linear regression models were tested post-hoc, with no significant heteroscedasticity (on Breusch-Pagan test) or autocorrelation of residuals present (on Durbin-Watson test). Small deviations from normality were present in the models’ residuals (Shapiro-Wilk test W > 0.98, p > 0.001). However, visual inspection of the Q–Q plot and histograms showed an approximately normal distribution with no strong skew or kurtosis. Given this and the large sample size (n > 400), the small deviation was deemed unlikely to impact the validity of the results.

### Association analyses with Q-CHAT scores – CHILD study

Regions showing non-significant trends towards association (at uncorrected p < 0.05) in the dHCP were further examined in the CHILD cohort, in which linear regression models were fitted including the same covariates as in the dHCP. Since segmentation methods of brain structure differed substantially between the cohorts, trending regions in the dHCP were broadly matched to regions in CHILD based on proximity (Supplementary Table 4). Regional analyses were further corrected via FDR to establish statistical significance (ROI of n = 15 regions for postnatal scans, n = 5 for fetal scans).

## Supplementary information

Supplementary Tables and Figures

## Data Availability

Structural imaging and behavioural traits of participants of the Developing Human Connectome (dHCP) can be obtained following an application. Details can be found online: https://biomedia.github.io/dHCP-release-notes/, with downloads completed via the NDA repository. https://nda.nih.gov/edit_collection.html?id=3955. Data of the CHILD study can be obtained following the signing of collaboration and data transfer agreements with the Autism Research Centre of the University of Cambridge.
