# Supplementary material for: Perinatal brain growth and autistic traits in toddlers
Source: Transl Psychiatry. 2025 Nov 17;15:474. doi: 10.1038/s41398-025-03665-0 (PMC12623961; doi:10.1038/s41398-025-03665-0)

Supplementary Material

Supplementary Figure 1: Autistic traits and prematurity. Only females (top panel) show a significant increase in Q-CHAT in extremely preterm cases (p-value is student's t-test).


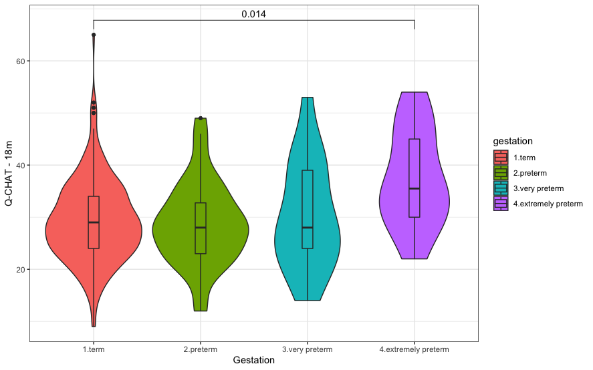

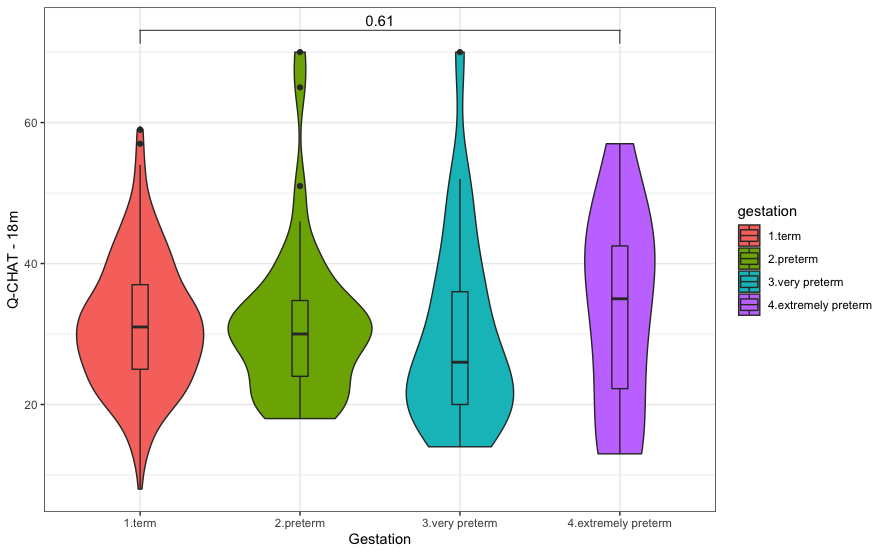


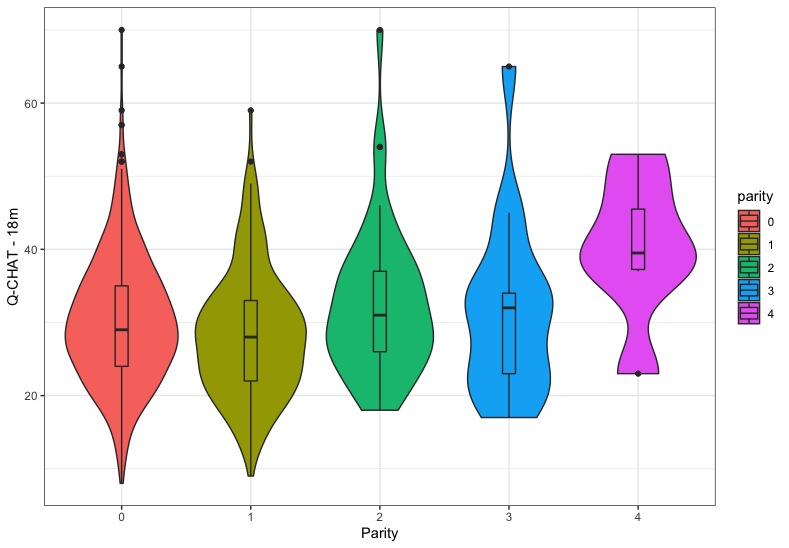


*Supplementary Figure 2: Autistic traits on Q-CHAT and maternal parity. No significant differences were found, when comparing pairwise or linearly via regression.*

*Supplementary Table 1: Parental and infant characteristics in the subset of the dHCP cohort in which brain volume was studied in association to Q-CHAT (n=454), based on specific exclusion criteria outlined in the main text.*

|  | Mean (SD) | Effect size for Q-CHAT | 95% CI | p - value |
| --- | --- | --- | --- | --- |
| **Maternal age** | 33.67 (4.74) | r=-0.142 | -0.231 – -0.051 | 0.002* |
| **Paternal age** | 36.16 (6.03) | r=-0.054 | -0.147 - 0.039 | 0.252 |
| **Maternal parity** | n-0=365  n-1=140  n-2=46  n-3=16  n-4=4  n-5=3 | r=-0.030 | -0.12 – 0.06 | 0.533 |
| **Gestational age at birth** | 39.61 (1.99)  weeks | r=-0.010 | -0.100- 0.085 | 0.879 |
| **Birth Weight** | 3.33 (0.59) kg | r=-0.05 | -0.140 – -0.045 | 0.305 |
| **Age at Q-CHAT** | 19.2(2.16) months | r=-0.58 | -0.149 - 0.034 | 0.217 |

*Supplementary Table 2: Maternal and infant characteristics in the CHILD cohort subset in which brain volume was studied in association to Q-CHAT (n=27).*

|  | Mean (SD) | Effect size for Q-CHAT | 95% CI | p - value |
| --- | --- | --- | --- | --- |
| **Maternal age** | 33.98 (2.7) | r=-0.104 | -0.495 – 0.322 | 0.637 |
| **Maternal parity** | n-0=15  n-1=6  n-2=3  n-3=0  n-4=1  unknown=2 | r=0.359 | -0.074 – 0.678 | 0.101 |
| **Gestational age at birth** | 39.58 (1.21) weeks | r=-0.245 | -0.59 – 0.18 | 0.248 |
| **Birth Weight** | 3.521 (4.65) kg | r=0.136 | -0.283 – 0.511 | 0.527 |
| **Age at Q-CHAT** | 80.342(2.78) weeks | r=-0.060 | -0.453 – 0.352 | 0.780 |

Model 2 identified regions that showed a non-significant trend towards interaction of total brain volume effect with Q-CHAT at an uncorrected level of significance, p<0.05 (Table 4). These included anterior cortical regions, such as the right anterior cingulate (both WM and GM), right anterior temporal lobe and structures such as the amygdala and the subthalamic nuclei bilaterally. Their interactions with TBV were negative, indicating that they were non-additive with TBV on Q-CHAT scores (Suppl Table 3). This was further supported by post-hoc categorisation based on TBV, with infants at the lower centiles showing positive trends for an association to Q-CHAT scores, compared to the higher TBV centiles (Suppl Figure 3).

*Suppl Table 3: Regions in the dHCP that showed a non-significant trend towards interaction with total brain volume in their association to Q-CHAT. None passed FDR-corrected of statistical significance. Model also included birth weight and age at Q-CHAT (data not shown, all non-significant)).r is partial coefficient of each variable in multiple regression.*

|  | dHCP Model 2 | Regional volume | TBV | Regional  x TBV | Sex | Maternal age |
| --- | --- | --- | --- | --- | --- | --- |
| GM | Anterior cingulate gyrus (R – GM) | r=0.112 | r=0.053 | r=-0.112 | r=0.117 | r=-0.124 |
|  |  | p=0.018 | p=0.268 | p=0.018 | p=0.013 | p=0.009 |
|  | Amygdala (R) | r=0.100 | r=0.074 | r=-0.111 | r=0.120 | r=-0.122 |
|  |  | p=0.035 | p=0.119 | p=0.019 | p=0.011 | p=0.010 |
|  | Subthalamic nucleus (L) | r=0.116 | r=0.047 | r=-0.100 | r=0.116 | r=-0.116 |
|  |  | p=0.015 | p=0.323 | p=0.035 | p=0.014 | p=0.015 |
|  | Subthalamic nucleus (R) | r=0.094 | r=0.057 | r=-0.098 | r=0.120 | r=-0.119 |
|  |  | p=0.047 | p=0.229 | p=0.039 | p=0.011 | p=0.012 |
| WM | Post. fusiform gyrus (L – WM) | r=0.102 | r=0.065 | r=-0.110 | r=0.122 | r=-0.121 |
|  |  | p=0.032 | p=0.170 | p=0.021 | p=0.010 | p=0.011 |
|  | Ant. cingulate gyrus (R – WM) | r=0.102 | r=0.058 | r=-0.106 | r=0.120 | r=-0.125 |
|  |  | p=0.032 | p=0.222 | p=0.025 | p=0.011 | p=0.008 |
|  | Thalamus (low intensity part - R) | r=0.122 | r=0.041 | r=-0.111 | r=0.112 | r=-0.115 |
|  |  | p=0.010 | p=0.382 | p=0.019 | p=0.018 | p=0.015 |
|  | Anterior lateral temporal lobe (R - WM) | r=0.095 | r=0.038 | r=-0.098 | r=0.116 | r=-0.115 |
|  |  | p=0.045 | p=0.422 | p=0.039 | p=0.014 | p=0.015 |


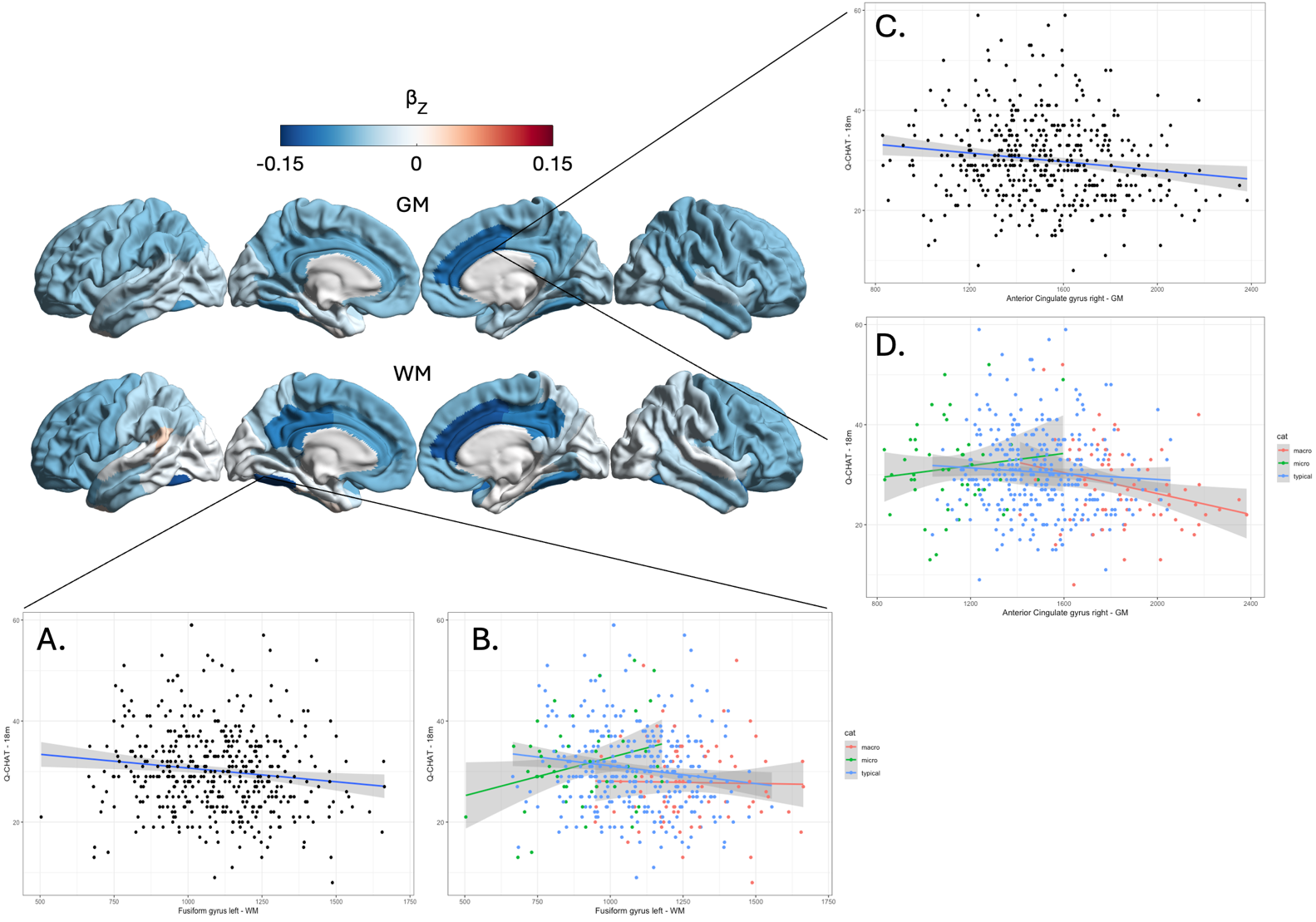


Suppl Figure 3: Atlas showing effect sizes of association to Q-CHAT of the interaction term that included postnatal total brain volume and the volumes of each cortical grey matter (GM) and white matter (WM) region in the dHCP, regardless of statistical significance. (A and C): Fusiform gyrus (left – WM) and anterior cingulate (right – GM) were associated negatively with Q-CHAT at a nominal level of statistical significance, (B and D): when considering TBV as a categorical variable, infants in lower centiles (category ‘micro’< (meanTBV- SD)) show evidence of positive associations, indicating an opposite non-significant interaction with TBV in predicting later autistic traits.

*Suppl Table 4: Matching of brain regions in the dHCP that were nominally associated with Q-CHAT scores, to regions that were studied in the CHILD ROI association analysis. Segmentation methods were based on the ‘drawEM’ template for dHCP and STA31 template for CHILD.*

| **Nominally associated brain segments in the dHCP with Q-CHAT scores** | **Equivalent regions for ROI in CHILD** |  |
| --- | --- | --- |
| Anterior lateral temporal lobe (R - WM) | Mid. Temporal Pole (R) |  |
|  |  |  |
|  | Sup. Temporal Pole (R) |  |
| Posterior medial-inferior temporal gyri (R – WM) | Mid. Temporal Gyrus (R) |  |
|  | Inf. Temporal Gyrus (R) |  |
| Amygdala (R) | Amygdala (R) |  |
|  |  |  |
| Posterior parahippocampal gyrus (L-GM) | Parahippocampal gyrus (L) |  |
| Posterior parahippocampal gyrus (R-WM) | Parahippocampal_R |  |
| Subthalamic nucleus (R) | Subthalamic Nucleus (R) |  |
| Anterior cingulate gyrus (R – GM) | Ant. Cingulum (R) |  |
| Anterior cingulate gyrus (R – WM) |  |  |
| Subthalamic nucleus (L) | Subthalamic Nucleus (L) |  |
| Thalamus (low intensity part - R) | Thalamus (R) |  |
|  |  |  |
| Thalamus (low intensity part - L) | Thalamus (L) |  |
|  |  |  |
| Lateral occipitotemporal & posterior fusiformis gyrus (L – WM)  Lateral occipitotemporal & anterior fusiformis gyrus (L - GM) | Fusiform Gyrus (L) |  |
| Lateral occipitotemporal & anterior fusiformis gyrus (R) | Fusiform Gyrus (R) |  |
| Frontal lobe (R-WM) | Frontal_Mid_R |  |
|  | Frontal_Sup_R |  |
| Brainstem spanning the midline | Midbrain_L |  |
|  | Midbrain_R |  |

*Supplementary Table 5: In CHILD, ROI analysis (Model 1) found no associations between the selected regions and autistic traits after controlling for TBV at either nominal or FDR-corrected level of significance.*

| Region | Regional volume | TBV | Sex | Maternal age | Age at Q-CHAT |
| --- | --- | --- | --- | --- | --- |
| Temporal_Pole_Sup_R | -0.443 | -0.242 | -0.074 | -0.185 | -0.443 |
|  | 0.075 | 0.349 | 0.778 | 0.478 | 0.075 |
| Frontal_Sup_R | -0.366 | 0.033 | -0.110 | -0.208 | -0.366 |
|  | 0.148 | 0.901 | 0.675 | 0.423 | 0.148 |
| Thalamus_R | -0.325 | -0.170 | 0.006 | -0.061 | -0.325 |
|  | 0.203 | 0.515 | 0.982 | 0.817 | 0.203 |
| Cingulum_Ant_R | 0.219 | -0.439 | -0.032 | -0.062 | 0.219 |
|  | 0.398 | 0.078 | 0.901 | 0.813 | 0.398 |
| Temporal_Mid_R | -0.217 | -0.243 | -0.030 | -0.118 | -0.217 |
|  | 0.402 | 0.347 | 0.910 | 0.653 | 0.402 |
| Temporal_Inf_R | -0.207 | -0.198 | -0.019 | -0.079 | -0.207 |
|  | 0.425 | 0.447 | 0.942 | 0.763 | 0.425 |
| Amygdala_R | 0.204 | -0.501 | -0.051 | -0.182 | 0.204 |
|  | 0.433 | 0.041 | 0.846 | 0.485 | 0.433 |
| Temporal_Pole_Mid_R | -0.177 | -0.409 | 0.037 | -0.190 | -0.177 |
|  | 0.496 | 0.103 | 0.889 | 0.466 | 0.496 |
| Thalamus_L | -0.108 | -0.328 | -0.018 | -0.127 | -0.108 |
|  | 0.681 | 0.198 | 0.946 | 0.626 | 0.681 |
| Frontal_Mid_R | 0.105 | -0.407 | -0.026 | -0.170 | 0.105 |
|  | 0.690 | 0.105 | 0.921 | 0.514 | 0.690 |
| Subthalamic_Nuc_R | -0.099 | -0.436 | -0.065 | -0.139 | -0.099 |
|  | 0.706 | 0.080 | 0.804 | 0.593 | 0.706 |
| Subthalamic_Nuc_L | -0.098 | -0.398 | -0.061 | -0.146 | -0.098 |
|  | 0.709 | 0.114 | 0.816 | 0.576 | 0.709 |
| ParaHippocampal_L | 0.074 | -0.410 | -0.043 | -0.164 | 0.074 |
|  | 0.778 | 0.102 | 0.870 | 0.528 | 0.778 |
| Fusiform_L | 0.069 | -0.371 | -0.056 | -0.165 | 0.069 |
|  | 0.793 | 0.143 | 0.831 | 0.526 | 0.793 |
| ParaHippocampal_R | 0.019 | -0.429 | -0.030 | -0.145 | 0.019 |
|  | 0.942 | 0.086 | 0.908 | 0.580 | 0.942 |

*Supplementary Table 6: Fetal global brain metrics of individuals scanned prenatally but not postnatally in the dHCP, and their association to Q-CHAT scores at 18 months. Additional covariates (data not shown) included fetal age at the time of scan in gestational weeks and birth weight.*

| Q-CHAT | TBV | Sex | Age at Q-CHAT | Maternal age | Age at scan |
| --- | --- | --- | --- | --- | --- |
| dHCP,  n=106 | r=-0.21 | r=0.23 | r=-0.10 | r=0.02 | r=0.20 |
|  | p=0.102 | p=0.076 | p=0.432 | p=0.864 | p=0.135 |
| Adjusted R-squared: 0.0367, p-value: 0.228 | | | | | |
| **Q-CHAT** | **Grey Matter** | **Sex** | **Age at Q-CHAT** | **Maternal age** | **Age at scan** |
| dHCP,  n=106 | r=-0.16 | r=0.20 | r=-0.10 | r=0.02 | r=0.13 |
|  | p=0.232 | p=0.123 | p=0.443 | p=0.899 | p=0.318 |
| Adjusted R-squared: 0.016, p-value: 0.335 | | | | | |
| **Q-CHAT** | **White Matter** | **Sex** | **Age at Q-CHAT** | **Maternal age** | **Age at scan** |
| dHCP,  n=106 | r=-0.15 | r=0.21 | r=-0.10 | r=-0.00 | r=0.13 |
|  | p=0.243 | p=0.110 | p=0.438 | p=0.972 | p=0.319 |
| Adjusted R-squared: 0.015, p-value: 0.342 | | | | | |
| **Q-CHAT** | **CSF** | **Sex** | **Age at Q-CHAT** | **Maternal age** | **Age at scan** |
| dHCP,  n=106 | r=-0.20 | r=0.17 | r=-0.13 | r=-0.01 | r=0.12 |
|  | p=0.123 | p=0.186 | p=0.313 | p=0.935 | p=0.358 |
| Adjusted R-squared: 0.032, p-value: 0.250 | | | | | |


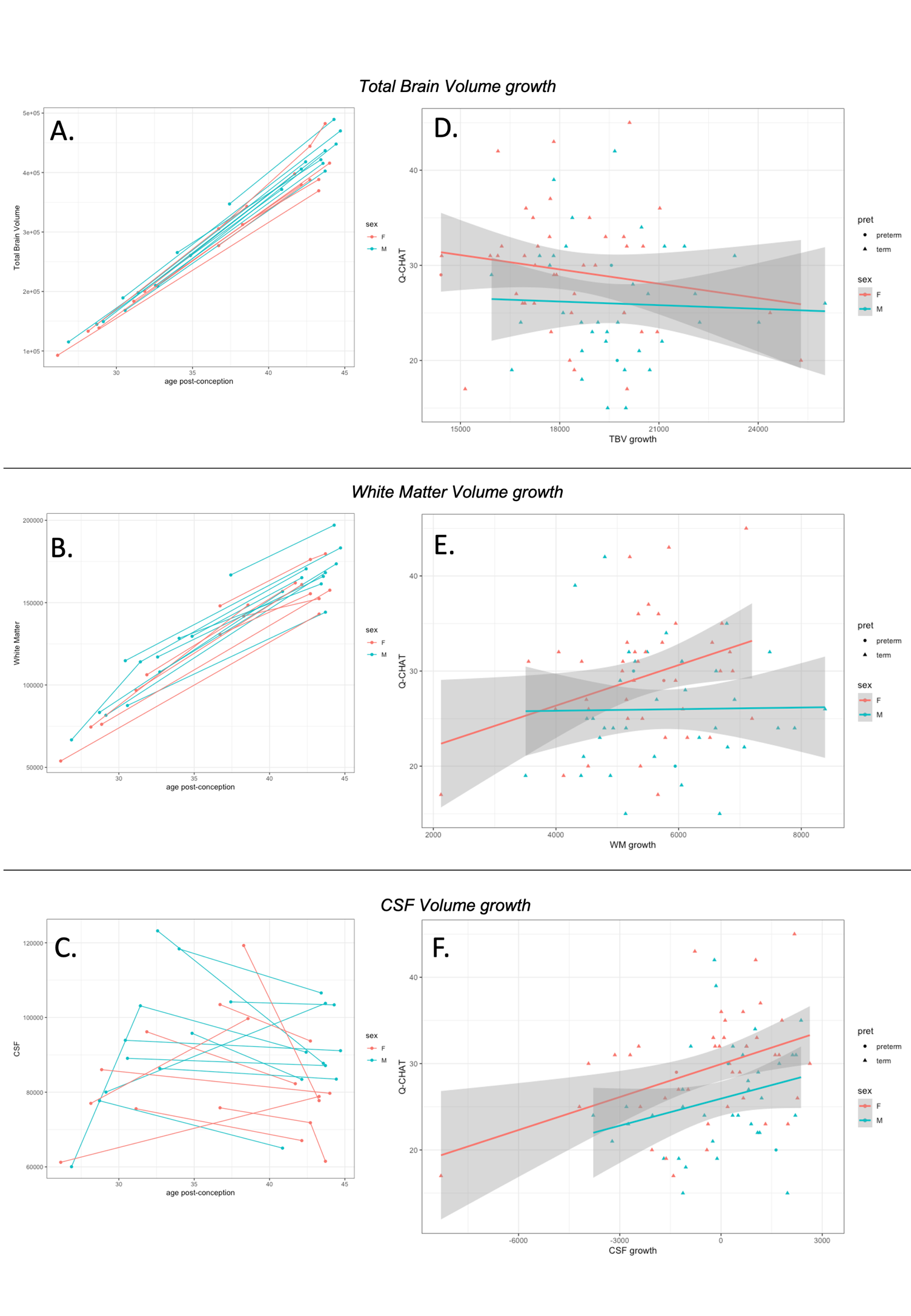


*Supplementary Figure 4: A-C: Linked total brain measurements for individuals scanned both prenatally and postnatally in the dHCP. D-F: composite rates of change in association with Q-CHAT scores for the same individuals.*

Supplementary Figure 5: Perinatal CSF change in CHILD and association with Q-CHAT.


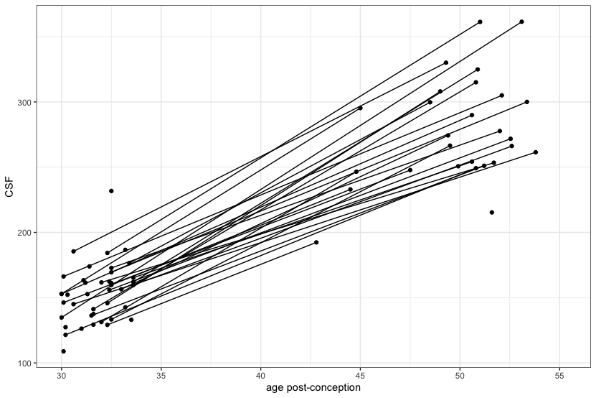

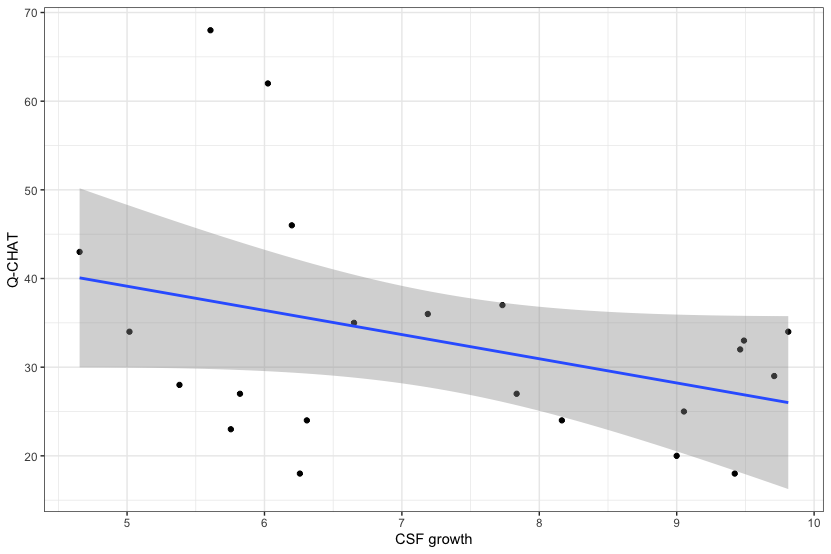

Supplement: Supplementary file 1 — Supplementary Tables and Figures [file 41398_2025_3665_MOESM1_ESM.docx]
